# Changing Practices of Opportunistic Salpingectomies and Oophorectomies From 1997 to 2017: A Nationwide Study

**DOI:** 10.1111/1471-0528.70183

**Published:** 2026-02-16

**Authors:** Jenni Hakkarainen, Aapeli Nevala, Nea Malila, Janne Pitkäniemi, Annika Auranen

**Affiliations:** ^1^ Finnish Cancer Registry Institute for Statistical and Epidemiological Cancer Research Helsinki Finland; ^2^ Faculty of Medicine and Health Technology (MET) Tampere University Tampere Finland; ^3^ Department of Obstetrics and Gynecology Kuopio University Hospital Kuopio Finland; ^4^ Department of Public Health, School of Medicine University of Helsinki Helsinki Finland; ^5^ Faculty of Social Sciences Tampere University Tampere Finland; ^6^ Department of Obstetrics and Gynecology Tampere University Hospital Tampere Finland; ^7^ Tays Cancer Centre Tampere University Hospital Tampere Finland

**Keywords:** epidemiology: general, gynaecology

In gynaecology, surgical removal of the ovaries (oophorectomy) and the Fallopian tubes (salpingectomy) has been performed as opportunistic procedures at the time of hysterectomy. Due to the vital role of ovaries in women's well‐being, the early 2000s saw a decrease in the use of opportunistic oophorectomy [1]. The incidence of opportunistic salpingectomy at the time of hysterectomy, however, has increased since the 2010s due to growing evidence of the tubal origin of high‐grade serous‐ovarian cancer justifying the removal of the Fallopian tubes for ovarian cancer prevention [2, 3, 4]. In recent decades, both globally and in Finland, the incidence of hysterectomy has undergone major changes [5], but while the proportion of salpingectomies performed during hysterectomy has increased, the actual population‐level incidence of salpingectomies remains largely unknown.

Therefore, to gain further insight into population level incidence, we examined national trends in the age‐standardised incidence (Finnish standard population 2015 per 10^5^ women‐years) of salpingectomies and oophorectomies in Finland over 20 years, from 1997 to 2017. We obtained data on hospital visits with adnexal procedure (Surgery involving the fallopian tubes and/or ovaries) from the Care Register for Health Care (HILMO), maintained by the Finnish Institute for Health and Welfare. We classified the procedures using the Nordic Classification of Surgical Procedures (NCPS). Bilateral salpingectomy codes were available from 2017 onward. Prior to 2017, it was possible to indicate bilaterality with a specific code for bilaterality; however, this practice was inconsistently implemented across hospitals. Therefore, when this specific code was not used, we interpreted salpingectomies at the time of hysterectomy as bilateral salpingectomies if there was no specific diagnosis code for tubal pathology. The study was approved by the Finnish Institute for Health and Welfare (Permit no. THL/367/6.02.00/2019; March 5, 2019).

From 1997 to 1999, the mean annual number of bilateral salpingo‐oophorectomies was 4113 (incidence 163.9/10^5^). The number decreased during 2006–2008 to 2442 (90.7/10^5^), then increased slightly in 2015–2017 to 2835 (98.5/10^5^) (Figure 1). At the beginning of the study period in 1997–1999, the mean annual number of bilateral salpingectomies was 766 (incidence 26.5/10^5^). The lowest mean annual number, 381 (13.6/10^5^), was observed in 2006–2008 followed by an increase to 1586 (59.3/10^5^) in 2015–2017. The median age at the time of any surgery, including bilateral salpingectomy, increased from 53.0 years in 1997–1999 to 55.1 in 2015–2017. The median age at the time of bilateral salpingectomy (without oophorectomy) decreased from 47.1 years in 1997–1999 to 45.8 years in 2015–2017. In 1997–1999, 36.2% of hysterectomies included salpingectomy (annually 4396 procedures), with the proportion increasing to 54.3% (3230 annual procedures) in 2015–2017. However, the total number of hysterectomies has decreased over time from 12 450 procedures in 1998 to 5991 in 2017 [5].

**FIGURE 1:**
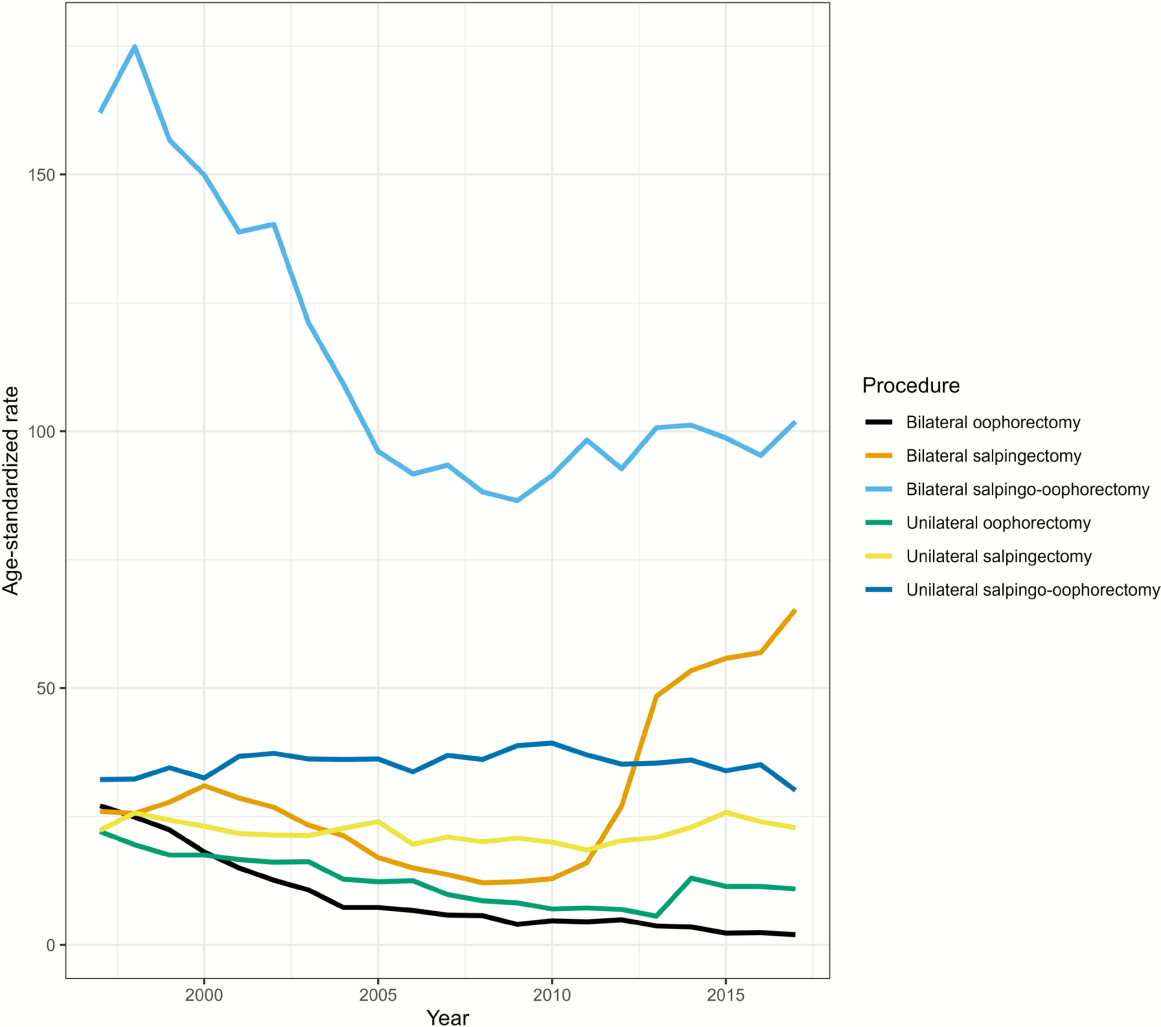
The incidence rate of oophorectomy and salpingectomy in Finland in 1997–2017.

Our evaluation of the Finnish registry data shows that the implementation of the current recommendations promoting opportunistic removal of the Fallopian tubes at the time of hysterectomy has been successful in Finland. Even so, the overall rate of salpingectomies was still lower in 2015–2017 compared to the beginning of the study period, explained by decreased overall incidence of hysterectomy during the study period. The lack of an unambiguous coding to separate bilateral and unilateral salpingectomy until 2017 is a limitation in this study, but we find our interpretation justified, based on national clinical coding practice. The main purpose of this study was to obtain the correct population‐at‐risk numbers for future estimation of ovarian cancer incidence, accounting for the surgical removal of the ovaries and the Fallopian tubes. Our ongoing research on ovarian cancer incidence in Finnish will examine whether surgery‐corrected incidence rates differ from the non‐corrected rates.

## Author Contributions

J.H. contributed to the data summary review and manuscript formation; J.H., A.A. and J.P. contributed to the planning of the study and the manuscript organisation; and A.N. contributed to the statistical programming and performed statistical analysis. All authors contributed to the manuscript preparation.

## Funding

Cancer Foundation Finland granted financial support for this research. J.H. has received a personal grant from the Finnish Society of Obstetrics and Gynecology.

## Conflicts of Interest

The authors declare no conflicts of interest.

## Data Availability

The data that support the findings of this study are available from the corresponding author upon reasonable request.
